# Siderophore-Microcins in *Escherichia coli*: Determinants of Digestive Colonization, the First Step Toward Virulence

**DOI:** 10.3389/fcimb.2020.00381

**Published:** 2020-08-21

**Authors:** Clémence Massip, Eric Oswald

**Affiliations:** ^1^IRSD, Université de Toulouse, INSERM, INRA, ENVT, UPS, Toulouse, France; ^2^Service de Bactériologie-Hygiène, Hôpital Purpan, CHU de Toulouse, Toulouse, France

**Keywords:** microcins, *Escherichia coli*, ExPEC, UPEC, genomic island (GI), intestinal colonization, pathogenesis, B2 phylogroup

## Abstract

Siderophore-microcins are antimicrobial peptides produced by enterobacteria, especially *Escherichia coli* and *Klebsiella pneumoniae* strains. The antibiotic peptide is post-translationally modified by the linkage of a siderophore moiety. Therefore, it can enter and kill phylogenetically related bacteria by a “Trojan Horse” stratagem, by mimicking the iron–siderophore complexes. Consequently, these antimicrobial peptides are key determinants of bacterial competition within the intestinal niche, which is the reservoir for pathogenic *E. coli*. The most frequent extraintestinal infections caused by *E. coli* are urinary tract infections. Uropathogenic *E. coli* (UPEC) can produce many virulence factors, including siderophore-microcins. Siderophore-microcins are chromosomally encoded by small genomic islands that exhibit conserved organization. In UPEC, the siderophore-microcin gene clusters and biosynthetic pathways differ from the “archetypal” models described in fecal strains. The gene cluster is shorter. Thus, active siderophore-microcin production requires proteins from two other genomic islands that also code for virulence factors. This functional and modular synergy confers a strong selective advantage for the domination of the colonic niche, which is the first step toward infection. This optimization of genetic resources might favor the selection of additional virulence factors, which are essential in the subsequent steps of pathogenesis in *E. coli* infection.

## Introduction

*Escherichia coli* lives as a commensal in the gut of warm-blooded animals (Tenaillon et al., 2010). It is also a major pathogen that causes intestinal and extraintestinal diseases, such as neonatal meningitis, nosocomial pneumonia, and bacteremia (Johnson and Russo, 2002; Kaper et al., 2004). The most common extraintestinal *E. coli* infections are urinary tract infections (UTIs) caused by uropathogenic *E. coli* (UPEC; Kaper et al., 2004; Flores-Mireles et al., 2015). UTIs are a serious public health issue in terms of morbidity, mortality, selection of antibiotic-resistant bacteria, and health care costs (Flores-Mireles et al., 2015).

*E. coli* strains are divided into eight phylogroups: A, B1, B2, C, D, E, F, and G (Tenaillon et al., 2010; Chaudhuri and Henderson, 2012; Clermont et al., 2019). Most extraintestinal pathogenic *E. coli*, including UPEC, belong to the *E. coli* phylogenetic group B2 (Picard et al., 1999; Johnson and Russo, 2002; Jaureguy et al., 2008; Tourret and Denamur, 2016). The reservoir of UPEC is gut microbiota, and *E. coli* from the B2 phylogroup are now the most frequently recovered in human fecal samples in industrialized countries (Touchon et al., 2009; Tenaillon et al., 2010). They produce more virulence factors than strains from other phylogroups (Picard et al., 1999), especially siderophore-microcins (Mcc; Budič et al., 2011; Micenková et al., 2016; Massip et al., 2020).

Mcc are antimicrobial peptides with a molecular mass below 10 kDa, produced by enterobacteria (Asensio et al., 1976; Pons et al., 2002). Siderophore-Mcc constitute a subclass of Mcc characterized by their molecular masses (between 5 and 10 kDa) and their post-translational modifications. They are post-translationally linked with a siderophore moiety derived from enterobactin (Azpiroz and Laviña, 2004; Thomas et al., 2004). Enterobactin is an iron uptake system produced by a non-ribosomal peptide synthetase (NRPS) pathway (for reviews, see Fischbach et al., 2006; Miethke and Marahiel, 2007).

Four siderophore-Mcc have been described so far: MccE492, MccH47, MccI47, and MccM. MccE492 is produced by *Klebsiella pneumoniae* and was first described in human fecal strain RYC492 (de Lorenzo and Pugsley, 1985). MccH47 was initially studied in human fecal strain *E. coli* H47 (Laviña et al., 1990). Like MccM, it is synthesized by *E. coli* fecal strains Nissle 1917, CA46, and CA58 (Davies and Reeves, 1975; Patzer, 2003; Vassiliadis et al., 2010) but also by many UPEC strains (Azpiroz et al., 2009; Šmajs et al., 2010; Abraham et al., 2012; Massip et al., 2020). MccI47 has only been mentioned in one study that focuses on the genetic systems that encode Mcc in *E. coli* strains H47 and CA46 (Poey et al., 2006).

In this review, we provide an overview of the current knowledge concerning siderophore-Mcc genetic systems and biosynthesis, their mechanisms of action, and their biological relevance in *E. coli*.

## Siderophore-Mcc Gene Clusters, Small Genomic Islands

The overall organization of siderophore-Mcc gene clusters is conserved. The G+C contents of siderophore-Mcc gene clusters are significantly lower (from 33 to 43%) than those of their host *E. coli* (51%) or *K. pneumoniae* (57.5%), which indicates that they might have been acquired from different bacteria by horizontal transfer (Duquesne et al., 2007). Moreover, siderophore-Mcc gene clusters are generally flanked by direct repeats described as acting as attachment sites in genomic islands (Azpiroz et al., 2011). Genomic islands are mobile genetic elements that help in the adaptation to a given environment. They contribute to the evolution of bacteria and undergo repeated rearrangements, deletions, and insertions (Dobrindt et al., 2010). Azpiroz et al. (2011) demonstrated that the MccH47 genetic system can be mobilized by site-specific recombination. Therefore, it was suggested that the siderophore-Mcc gene cluster is a small genomic island (Azpiroz et al., 2011).

**Figure 1:**
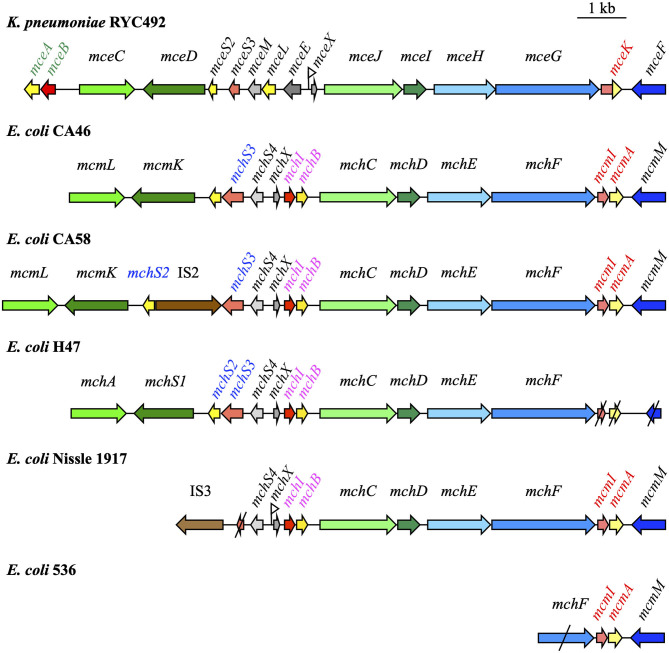
Microcin (Mcc) gene clusters in *Klebsiella pneumoniae* RYC492, *Escherichia coli* CA46, CA58, H47, Nissle 1917, and 536 [modified from Massip et al. (2019)]. Mcc precursor genes, immunity genes, and genes involved in Mcc export and post-translational modifications are indicated in yellow, red, blue, and green, respectively. Truncated genes are shown with slashes. The names of the genes specifically involved in MccE492, MccM, MccH47, and MccI47 are in green, red, pink, and blue, respectively. Fur boxes proved important in the regulation of Mcc production are marked by a small flag.

Genes responsible for siderophore-Mcc biosynthesis and export are gathered in large clusters (up to 13 kb) on the bacterial chromosome (Figure 1). The minimal structure comprises the gene that encodes the Mcc precursor peptide and the self-immunity gene, which are grouped in an operon, and genes responsible for the export of the siderophore-Mcc. Genes *mceA, mchB, mchS2*, and *mcmA* encode precursor peptides of 103, 75, 77, and 92 amino acids for MccE492, MccH47, MccI47, and MccM, respectively (Gaggero et al., 1993; Lagos et al., 2001; Poey et al., 2006; Duquesne et al., 2007). The mechanism of self-immunity to siderophore-Mcc remains largely elusive. MccE492, MccH47, MccI47, and MccM have specific self-immunity proteins. Studies of their sequence indicate that they are probably membrane-bound, with two or three transmembrane helices (Rodríguez et al., 1999; Lagos et al., 2001; Duquesne et al., 2007).

In general, the siderophore-Mcc gene cluster also bears genes that encode post-translational modifications enzymes (Poey et al., 2006; Duquesne et al., 2007). When several siderophore-Mcc are encoded by a single gene cluster (e.g., MccH47 and MccM in *E. coli* Nissle), genes responsible for post-translational modifications and export can be shared (Poey et al., 2006). Genes involved in post-translational modifications *mceC, mchA*, and *mcmL* are homologous to *iroB* encoding a glycosyltransferase from the salmochelin genetic system *iroA* (Lagos et al., 2001; Patzer, 2003). Salmochelins are siderophores derived from enterobactin through glycosylation (Fischbach et al., 2006). Similarly, *mceD, mchS1*, and *mcmK* are homologous to *iroD* encoding an enterobactin esterase (Lagos et al., 2001; Patzer, 2003). Proteins MceI, MceJ, and their homologs MchC, MchD link the Mcc precursor peptides with the siderophore moiety derived from enterobactin, but the precise function of these proteins remains unknown (Nolan and Walsh, 2008).

Siderophore-Mcc are transported outside the producing bacteria by a three-component ATP-binding cassette (ABC) exporter. Two components are encoded as an operon on the Mcc gene cluster: the ABC transporter protein (MchF for MccH47 and MccM, MceG for MccE492) and the accessory protein of the membrane fusion protein (MFP) family (MchE for MccH47 and MccM, MceH for MccE492). These proteins are highly similar with 92 and 94% identical amino acids between the two types of ABC and MFP proteins, respectively. The third component of this export system is the outer membrane protein TolC (Azpiroz et al., 2001; Lagos et al., 2001; Patzer, 2003).

Although the general organization of the siderophore-Mcc island is conserved, substantial differences can be found between strains. Traces of recombination events can be detected at the center of siderophore-Mcc gene clusters. In *E. coli* strain CA58, genes encoding transposases are inserted between genes *mchS2* and *mchS3* encoding MccI47 precursor and immunity peptides. Since these two genes are normally organized in an operon, the translation process is compromised, at least for *mchS2*, which is downstream of the insertion sequence. Consequently, unlike strain CA46, *E. coli* CA58 does not produce MccI47 (Patzer, 2003; Poey et al., 2006; Vassiliadis et al., 2010).

In UPEC strain 536, the siderophore-Mcc island has undergone considerable changes if strain CA46 is considered as a reference. Only four genes remain: genes encoding MccM precursor and immunity peptides, and two genes involved in Mcc export, *mcmM* and *mchF*, which are truncated. In the absence of a functional export system (association between ABC and MFP proteins), strain 536 might not be able to secrete siderophore-Mcc.

Finally, in *E. coli* Nissle and in the UPEC strains CFT073 and ABU83972, the island is truncated in the 5′ region. Compared to the strain CA46 island, it is deprived of the MccI47 precursor peptide and self-immunity genes, as well as post-translational modification genes *mcmL* and *mcmK* (Patzer, 2003; Poey et al., 2006). In strains bearing such a truncated island, genes encoding transposases flank the 5′ region as remnants of recombination events (Patzer, 2003). In the three *E. coli* strains Nissle, CFT073, and ABU83972, the truncated Mcc gene cluster is located on the genomic island I that covers the *serX* tRNA locus [for reviews, see Dobrindt et al. (2004, 2010)]. This genomic island also carries the *iroA* locus with the *mcmL* and *mcmK* homologs *iroB* and *iroD*. It is highly conserved in the three strains and in the Mcc gene cluster in particular, with 100% similarity (Grozdanov et al., 2004; Vejborg et al., 2010; Reister et al., 2014). Therefore, Poey et al. (2006) suggested considering the siderophore-Mcc gene cluster as “an islet inside an island.” It provides a selective advantage, is acquired by horizontal transfer, and can undergo significant rearrangements.

## Biosynthesis of Siderophore-Mcc and Mode of Action

**Figure 2:**
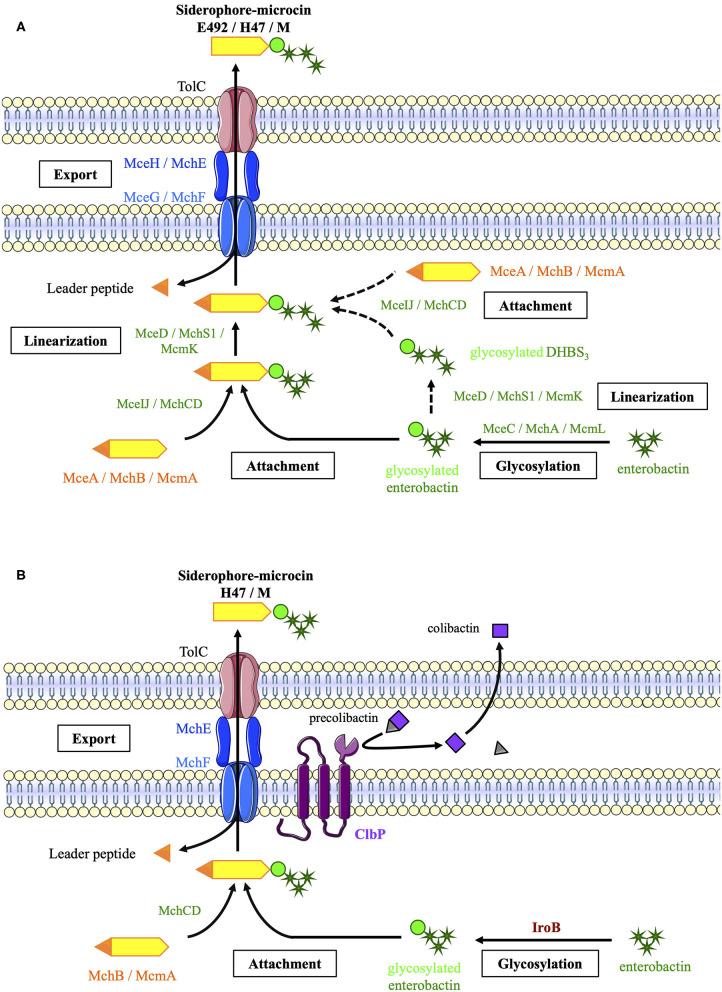
Biosynthetic pathways of siderophore-microcins (Mcc) **(A)** in strains carrying an “archetypal Mcc gene cluster” with genes encoding the enterobactin esterase and glycosyltransferase **(B)** in *E. coli* strains from the B2 phylogroup carrying a “truncated Mcc gene cluster.” This biosynthesis requires the glycosyltransferase IroB encoded by the salmochelin gene cluster and the ClbP protein encoded by the *pks* island, which are indicated in brown and purple, respectively [adapted from Massip et al. (2019)]. Mcc precursor peptides and proteins involved in Mcc export and post-translational modifications are indicated in orange, blue, and green, respectively.

Siderophore-Mcc biosynthesis begins with the production of two separate moieties: (1) a precursor peptide encoded by the Mcc island and (2) enterobactin by an NRPS pathway (Figure 2A). Glycosyltransferases MceC, MchA, or McmL catalyze the transfer of one glucose to the 2,3-dihydroxybenzoic acid moieties of enterobactin (Nolan et al., 2007; Vassiliadis et al., 2007). In fact, unlike its homolog IroB, the catalytic efficiency of MceC decreases with enterobactin glycosylation (Nolan et al., 2007). MceIJ or their homolog MchCD then links the C-glycosylated enterobactin to the serine and glycine-rich C-terminal region of the precursor peptide (Nolan and Walsh, 2008; Vassiliadis et al., 2010). The enterobactin esterase MceD, MchS1, or McmK hydrolyzes the C-glycosylated enterobactin into a glycosylated linear trimer of 2,3-dihydroxybenzoyl-serine (Nolan et al., 2007; Vassiliadis et al., 2007). Alternatively, the glycosylated enterobactin could be linearized prior to its linkage to the precursor peptide. None of these pathways could be excluded in *in vitro* MccE492 synthesis experiments. MceIJ complex catalyzes the attachment of glycosylated enterobactin, whether or not it is linearized, and MceD can hydrolyze glycosylated enterobactin whether or not it is linked to the precursor peptide (Nolan et al., 2007; Nolan and Walsh, 2008). Enterobactin glycosylation is catalyzed before linearization, because the catalytic efficiency of MceC decreases with the linearization of the enterobactin scaffold (Nolan et al., 2007).

The last step of the siderophore-Mcc maturation process is concomitant with export. Siderophore-Mcc precursors carry an N-terminal leader peptide of 15–19 amino acids, which probably acts as a recognition signal for the ABC transporter (MceG or MchF; Pons et al., 2002; Vassiliadis et al., 2010). The ABC transporter N-terminal domain with a protease activity cleaves the leader peptide during export (Azpiroz et al., 2001; Vassiliadis et al., 2010). The precise role of the accessory protein (MceH or MchE) has not been examined. However, it may act as a connector between the ABC transporter and the outer membrane protein TolC (Pons et al., 2002; Duquesne et al., 2007).

In strains such as *E. coli* Nissle, deprived of *mcmL* and *mcmK*, the siderophore-Mcc synthesis pathway is slightly different (Figure 2B). The enterobactin moiety is glycosylated by IroB from the salmochelin pathway instead of the missing McmL. Moreover, the periplasmic protein ClbP involved in colibactin synthesis (Nougayrède et al., 2006) is essential for MccH47 and MccM production (Massip et al., 2019). Even if the exact production pathway in such strains has not been elucidated, the C-terminal domain of ClbP has been suggested to facilitate the export of MccH47 and MccM, in which enterobactin moiety would not be linearized in the absence of the esterase McmK (Massip et al., 2019).

**Figure 3:**
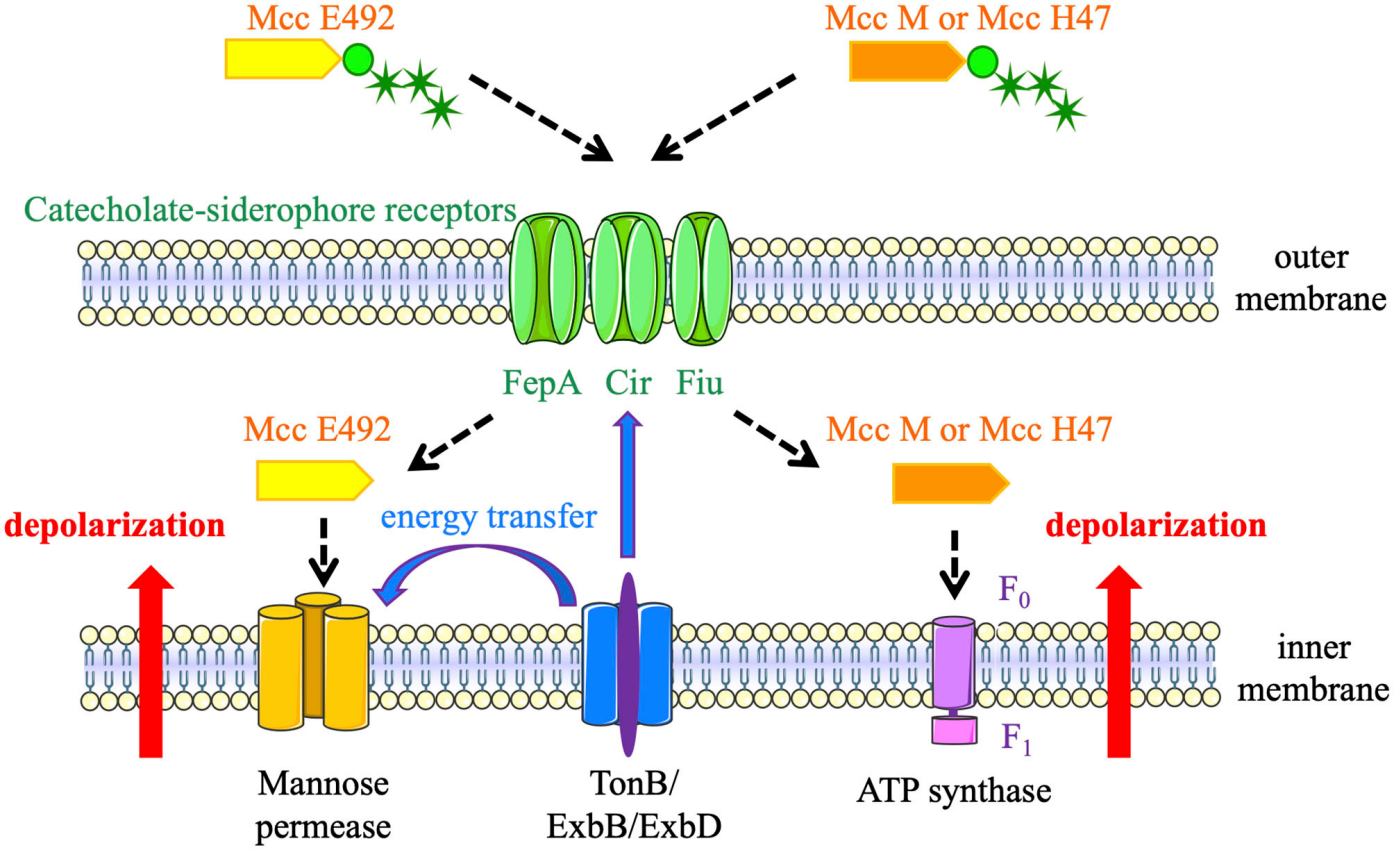
Siderophore-microcins (Mcc) mechanisms of action. MccE492, MccH47, and MccM enter target bacteria by catecholate siderophore receptors (FepA, Fiu, or Cir). MccE492 interacts with the mannose permease, whereas MccH47 and MccM target the F_0_ proton channel of the ATP synthase. These interactions trigger inner-membrane depolarization and cell death. The energy produced by the TonB machinery is required for translocation of the three siderophore-Mcc and the formation of channels or pores by MccE492 linkage with the mannose permease.

Siderophore-Mcc were labeled “Trojan Horse” toxins because they enter target bacteria by the catecholate siderophore receptors FepA, Fiu, or Cir (Figure 3), thanks to their post-translational modification with an enterobactin moiety (Patzer, 2003; Thomas et al., 2004; Vassiliadis et al., 2010). Consequently, they target enterobacteria that are phylogenetically similar and can be competitors for space and resources in a given niche. Once recognized by siderophore receptors, siderophore-Mcc are translocated through the outer membrane using the energy produced by the TonB machinery (Destoumieux-Garzón et al., 2003). Once in the periplasm, MccE492 interacts with the ManY/ManZ inner-membrane components of the mannose permease. It triggers channel or pore formation and TonB-dependent inner-membrane depolarization, followed by cell death (Bieler et al., 2006). MccH47 has a different cellular target: the ATP synthase (Trujillo et al., 2001). To be more precise, the F_0_ proton channel is required for MccH47 activity. MccH47 provokes an unregulated entry of protons, which dissipates the membrane potential (Rodriguez and Lavina, 2003). The mechanism of action of MccM has never been established, but it is suspected to be the same as that of MccH47.

Siderophore-Mcc production is highly regulated by iron concentration through the ferric uptake regulator (Fur) protein (Patzer, 2003; Vassiliadis et al., 2007). This protein acts as a transcriptional repressor and senses intracellular iron availability (Bagg and Neilands, 1987). Fur boxes are located upstream of *mceX* and *mchX* genes (Patzer, 2003; Vassiliadis et al., 2007). At high iron availability, Fur–Fe^2+^ complexes repress *mceX*, in which transcription is coupled with *mceIJ* genes. Therefore, MceIJ cannot catalyze the attachment of the enterobactin moiety with the MccE492 precursor. Moreover, MceX acts as a negative regulator of *mceA* and *mceB* genes. Therefore, a high iron concentration allows a high expression of the MccE492 precursor, leading to a predominant synthesis of unmodified Mcc (Marcoleta et al., 2018). This form has a low antibacterial activity because it is not recognized by siderophore receptors (Thomas et al., 2004).

## Impacts of Siderophore-Mcc Produced by *E. coli*

*E. coli* causes various extraintestinal infections, of which UTIs are the most frequent. *E. coli* is also a frequent cause of neonatal meningitis, bacteremia, and nosocomial infections such as nosocomial pneumonia (Johnson and Russo, 2002; Kaper et al., 2004). The patient's own intestinal flora is the reservoir for these so-called extraintestinal pathogenic *E. coli* (Russo and Johnson, 2000; Starčič Erjavec and Žgur-Bertok, 2015), especially for UPEC (Yamamoto et al., 1997; Moreno et al., 2008). A UPEC strain residing in the gut emerges from the rectal flora, colonizes the perineal region and the urethra, and then migrates to the bladder (Flores-Mireles et al., 2015). Therefore, effective intestinal colonization is a prerequisite for UTI development.

In industrialized countries, most human fecal strains belong to the B2 phylogroup, whereas most strains from Africa, Asia, or South America belong to the A phylogroup (Gordon and O'Brien, 2006; Tenaillon et al., 2010). The differences in phylogroup distribution are probably linked to socioeconomic factors, such as dietary and hygiene habits, which condition the gut microbiota (Tenaillon et al., 2010). A 1-year study in which the fecal strains of Swedish infants were monitored revealed that *E. coli* from the B2 phylogroup had a capacity for enhanced persistence in their intestinal microflora (Nowrouzian et al., 2005). Studies of fecal *E. coli* in Australia and the Czech Republic revealed that MccH47- and MccM-producing strains are overrepresented in the B2 phylogroup (Gordon and O'Brien, 2006; Micenková et al., 2016). Since *E. coli* siderophore-Mcc target phylogenetically related *Enterobacteriaceae*, they probably confer a selective advantage in the intestinal niche.

The proportion of B2 phylogroup strains seems to be higher in UPEC than in fecal strains: 72 and 69% in UPEC (Abraham et al., 2012; Massip et al., 2020), and 45 and 30% in fecal strains (Tenaillon et al., 2010) in Australia and France, respectively. Similarly, siderophore-Mcc-producing strains represent ~30% of UPEC (Šmajs et al., 2010; Abraham et al., 2012; Massip et al., 2020), compared to ~15% of fecal strains (Gordon and O'Brien, 2006; Micenková et al., 2016). Like in fecal *E. coli*, there are significantly more UPEC strains carrying siderophore-Mcc genes among B2 phylogroup strains than among non-B2 strains (Azpiroz et al., 2009; Abraham et al., 2012; Massip et al., 2020).

Most UPEC strains that produce siderophore-Mcc carry a “truncated” Mcc gene cluster deprived of genes *mcmL* and *mcmK*. Besides very rare exceptions, they also bear the *pks* and salmochelin islands (Azpiroz et al., 2009; Massip et al., 2020), which correspond with the functional synergy recently demonstrated between these three gene clusters used to synthetize MccH47 and MccM (Massip et al., 2019). The triple combination of “truncated” Mcc gene cluster, *pks*, and salmochelin islands enables the production of at least five virulence factors in addition to Mcc: the siderophores enterobactin, salmochelins, and yersiniabactin (Martin et al., 2013), colibactin (Nougayrède et al., 2006), and analgesic lipopeptides (Pérez-Berezo et al., 2017). The versatility of these three genomic islands leads to “genomic economy.” At least three pleiotropic proteins are encoded by this triad: IroB, which intervenes in Mcc and salmochelins syntheses (Fischbach et al., 2005; Massip et al., 2019); ClbA, which triggers colibactin, analgesic lipopeptide, and enterobactin production (Nougayrède et al., 2006; Martin et al., 2013; Pérez-Berezo et al., 2017); and ClbP, which is involved in Mcc and colibactin pathways (Dubois et al., 2011; Massip et al., 2019). Therefore, this type of *E. coli* strain can adapt to various environments with a limited impact on genome size. They persist and emerge from the polymicrobial intestinal niche, adapt to a nutrient-poor environment, and evade the host defense system.

Although this triple combination is frequent among UPEC, it is not correlated with the clinical severity of the infection (Abraham et al., 2012; Massip et al., 2020). Thus, it might not be a virulence factor *per se* within the urinary tract. However, a transcriptional analysis of the pyelonephritis strain CFT073 grown in urine revealed that *mchB* and *iroB* are among the 50 most upregulated genes (Snyder et al., 2004). A similar study performed with the asymptomatic bacteriuria strain ABU83972 and the probiotic strain Nissle, in addition to CFT073, showed that microcin, colibactin, and salmochelin genes are induced when the strains are grown in urine (Hancock et al., 2010). Considering that MccH47 and MccM are the most frequent Mcc in UPEC (Šmajs et al., 2010; Abraham et al., 2012), it suggests that the synergistic triad between microcin, colibactin, and salmochelin islands could promote urinary tract colonization (Massip et al., 2020). This hypothesis is supported by a study that compared the whole genome sequences of UTI and fecal isolates from the same patients. The microcin M activity protein (McmM) was significantly overrepresented in UTI isolates compared to strictly fecal isolates, unlike the classical UPEC virulence factors (e.g., fimbriae; Nielsen et al., 2017).

Moreover, UPEC strains that carry MccM and MccH47 determinants possess a greater number of other virulence factors (e.g., *hlyA* and *cnf1*) than isolates deprived of these siderophore-Mcc genes (Abraham et al., 2012; Massip et al., 2020). It suggests that the triad of the truncated Mcc gene cluster, the *pks* island, and the *iroA* locus enables the domination of the rectal niche with minimal genetic cost, which might favor the selection of additional virulence factors. The accumulation of these virulence factors eventually determines the strain pathogenicity level in a given host urinary tract.

In conclusion, MccH47 and MccM seem to be key determinants for effective intestinal colonization by B2 phylogroup *E. coli*. They might be particularly important in UPEC strains, for which domination and emergence from the rectal reservoir is a prerequisite for urinary tract colonization. Their production in B2 phylogroup strains seems to be optimized to minimize genetic cost and allow further selection of virulence factors. Hence, siderophore-Mcc could be a cornerstone of extraintestinal pathogenic *E. coli* versatility.

## Author Contributions

CM wrote the first draft of the manuscript. Both authors contributed to manuscript revision, read, and approved the submitted version.

## Conflict of Interest

The authors declare that the research was conducted in the absence of any commercial or financial relationships that could be construed as a potential conflict of interest.
